# Pain drawings as a diagnostic tool for the differentiation between two pain-associated rare diseases (Ehlers-Danlos-Syndrome, Guillain-Barré-Syndrome)

**DOI:** 10.1186/s13023-020-01542-1

**Published:** 2020-11-17

**Authors:** Larissa Wester, Martin Mücke, Tim Theodor Albert Bender, Julia Sellin, Frank Klawonn, Rupert Conrad, Natasza Szczypien

**Affiliations:** 1 Center for Rare Diseases Bonn (ZSEB), University Hospital Bonn, Bonn, Germany; 2 Institute for Information Engineering, Ostfalia University of Applied Sciences, Wolfenbüttel, Germany; 3 Biostatistics Group, Helmholtz Centre for Infection Research, Braunschweig, Germany; 4 Department of Psychosomatic Medicine and Psychotherapy, University Hospital Bonn, Bonn, Germany

**Keywords:** Differential diagnosis, Ružička similarity, Orphan diseases, Self-report instrument, ORPHA: 98249, ORPHA: 2103

## Abstract

**Background:**

The diagnosis of rare diseases poses a particular challenge to clinicians. This study analyzes whether patients’ pain drawings (PDs) help in the differentiation of two pain-associated rare diseases, Ehlers-Danlos Syndrome (EDS) and Guillain-Barré Syndrome (GBS).

**Method:**

The study was designed as a prospective, observational, single-center study. The sample comprised 60 patients with EDS (3 male, 52 female, 5 without gender information; 39.2 ± 11.4 years) and 32 patients with GBS (10 male, 20 female, 2 without gender information; 50.5 ± 13.7 years). Patients marked areas afflicted by pain on a sketch of a human body with anterior, posterior, and lateral views. PDs were electronically scanned and processed. Each PD was classified based on the Ružička similarity to the EDS and the GBS averaged image (pain profile) in a leave-one-out cross validation approach. A receiver operating characteristic (ROC) curve was plotted.

**Results:**

60–80% of EDS patients marked the vertebral column with the neck and the tailbone and the knee joints as pain areas, 40–50% the shoulder-region, the elbows and the thumb saddle joint. 60–70% of GBS patients marked the dorsal and plantar side of the feet as pain areas, 40–50% the palmar side of the fingertips, the dorsal side of the left palm and the tailbone. 86% of the EDS patients and 96% of the GBS patients were correctly identified by computing the Ružička similarity. The ROC curve yielded an excellent area under the curve value of 0.95.

**Conclusion:**

PDs are a useful and economic tool to differentiate between GBS and EDS. Further studies should investigate its usefulness in the diagnosis of other pain-associated rare diseases. This study was registered in the German Clinical Trials Register, No. DRKS00014777 (Deutsches Register klinischer Studien, DRKS), on 01.06.2018.

## Background

Rare diseases present a great challenge for patients and physicians. The long way of suffering most patients must go through before they receive the correct diagnosis and treatment not only leads to enormous mental, physical, and social distress, but also places high economic burdens on our health care system [1, 2].

The rareness and diversity of orphan diseases corresponds to a lack of knowledge among health care professionals, and both factors impede diagnosis and therapy [3, 4]. Often, an endless number of medical consultations is necessary, and many years pass by until a patient discovers their diagnosis.

Against this backdrop the current study aims to explore the utility of patients’ pain drawings as a novel diagnostic tool in the differentiation and diagnosis of two rare diseases, Ehlers-Danlos Syndrome (EDS) and Guillain-Barré Syndrome (GBS). This methodological approach is based on the idea that repeating patterns in pain drawings (PDs) could help to accelerate diagnostic proceedings and sensitize physicians for rare diseases. The concept of pain drawing was implemented in 1949 by Palmer [5] and since then, PDs have been used as a diagnostic tool. Patients are asked to mark painful regions in a simple line drawing of the human body, which may even be more precise compared to the verbal description of pain symptoms, particularly in patients with difficulties in verbal fluency. To date, a few studies have shown that PDs can be used as a screening tool for different purposes. Rennerfelt et al. showed that PDs might be a valuable instrument in diagnosing the causes of exercise-induced leg pain. Based on PDs, two observers correctly identified patients with and without chronic anterior compartment syndrome (CACS), which is the most common cause of exercise-induced leg pain. The test–retest showed a high inter-observer agreement of 84% [6]. Abott et al. proved that specific Pain Drawing Scores [using the Simple Body Region (SBR) method and the Pain Sites Score (PSS)] in patients with recurrent or chronic low back pain (RCLBP) predict an increased risk of depression, somatization and distress at 1-year follow-up [7].

For practical reasons regarding availability of patients, we chose EDS and GBS as two different disorders representing rare diseases associated with pain as primary symptom. EDS includes a heterogeneous group of inherited disorders, which are caused by different mutations in genes encoding fibrillar collagen or collagen-modifying enzymes [8, 9]. The prevalence ranges from 1:150,000 to 1:5,000 depending on the population [10]. The defective collagen causes fragility of the soft connective tissues and extensive manifestations in skin, ligaments and joints, blood vessels, and internal organs. The clinical spectrum varies from mild skin hyperextensibility, joint hypermobility, and tissue fragility, to severe physical disability and life-threatening vascular complications. In most of the cases, pain is the first clinical symptom. EDS induces mild to severe pain. 90% of the affected patients suffer from chronic pain [11]. The current Villefranche classification recognizes six subtypes, while more recent studies describe 13 variants [8]. Since there is no curative treatment for any type of EDS, early diagnosis is of prime importance in order to optimize the symptomatic management of patients and to prevent avoidable complications [12]. GBS is an acute postinfectious polyradiculoneuropathy with a variable clinical presentation. Every year, 1.1 to 1.8 per 100,000 persons suffer from GBS [13]. It is caused by autoantibodies that attack peripheral nerve components [14]. The symptoms range from ascending bilateral limb weakness to decreased reflexes and severe back or extremity pain [15, 16]. Pain can be seen as an heralding feature and can remain for 2 years [17]. GBS can lead to respiratory insufficiency making an early diagnosis and the initiation of an appropriate treatment essential.

## Results

### Pain questionnaire

While PDs are valuable tools to communicate which part of the body is affected, they however do not contain information about quality and intensity of pain. Therefore, patients also filled in a pain questionnaire based on the “painDETECT questionnaire” and the “German pain questionnaire” in order to obtain information with regards to the quality of the pain experienced. This information was gathered in order to complement the regional information obtained from the PDs, and to have a data basis for future endeavors to combine PDs and questionnaire results into a single tool. The results are summarized in Table 1.

**Table 1:** Summary of pain questionnaire answers and marked areas in PDs. n is number of people answering the question. Percentages indicate the % of people asked (% of n) that gave the indicated answer. Average value refers to the scale used for some questions (0 = not at all, 3 = high intensity). Bold type indicates most common answers. The summary of the pain profiles groups the marked areas from most common to least common, with the percentage of PDs indicating the mentioned area (% of PDs column)

GBS		EDS	
**Possible answers**	**% Answered (n)**	**Possible answers**	**% Answered (n)**
1. Pain duration			
Less than 1 month	3.10% (n = 32)	Less than 1 month	0% (n = 60)
1 month to 1/2 year	6.30% (n = 32)	1 month to 1/2 year	0% (n = 60)
1/2 year to 1 year	6.30% (n = 32)	1/2 year to 1 year	0% (n = 60)
1 to 2 years	**21**.**90% (n** = **32)**	1 to 2 years	3.3% (n = 60)
2 to 5 years	**28**.**10% (n** = **32)**	2 to 5 years	6.7% (n = 60)
More than 5 years	25.00% (n = 32)	More than 5 years	**86**.**7% (n** = **60)**
2. Pain perception			
Persistent pain with light fluctuations	**25**.**00% (n** = **32)**	Persistent pain with light fluctuations	11.7% (n = 60)
Persistent pain with severe fluctuations	6.30% (n = 32)	Persistent pain with severe fluctuations	**28**.**3% (n** = **60)**
Pain attacks without pain in between	**34**.**40% (n** = **32)**	Pain attacks without pain in between	11.7% (n = 60)
Pain attacks with pain in between	21.90% (n = 32)	Pain attacks with pain in between	**45% (n** = **60)**
3. Pain attacks			
3a. Average frequency			
Several times a day	**28**.**10% (n** = **32)**	Several times a day	**20% (n** = **60)**
Daily	6.30% (n = 32)	Daily	8.3% (n = 60)
Several times a week	**18**.**80% (n** = **32)**	Several times a week	**18**.**3% (n** = **60)**
Weekly	0.00% (n = 32)	Weekly	1.7% (n = 60)
Several times a month	6.30% (n = 32)	Several times a month	10% (n = 60)
Monthly	3.10% (n = 32)	Monthly	0% (n = 60)
3b. Average duration			
Less often	0.00% (n = 32)	Less often	1.7% (n = 60)
Seconds	3.10% (n = 32)	Seconds	1.7% (n = 60)
Minutes	**15**.**60% (n** = **32)**	Minutes	6.7% (n = 60)
Hours	**28**.**10% (n** = **32)**	Hours	**28**.**3% (n** = **60)**
Up to 3 days	9.40% (n = 32)	Up to 3 days	**11**.**7% (n** = **60)**
Longer than 3 days	3.10% (n = 32)	Longer than 3 days	10% (n = 60)
3c. Particularly severe pain at a certain daytime			
No	**25**.**00% (n** = **32)**	No	**28**.**3% (n** = **60)**
Yes, in the morning	3.10% (n = 32)	Yes, in the morning	11.7% (n = 60)
Yes, at noon	3.10% (n = 32)	Yes, at noon	0% (n = 60)
Yes, in the afternoon	3.10% (n = 32)	Yes, in the afternoon	5% (n = 60)
Yes, in the evening	**15**.**60% (n** = **32)**	Yes, in the evening	**16**.**7% (n** = **60)**
Yes, at night	12.50% (n = 32)	Yes, at night	5% (n = 60)
4. Pain sensation			
4a. Physical pain qualities			
**Pain quality**	**% answered “medium to high intensity” (n, average value)**	**Pain quality**	**% answered “medium to high intensity” (n, average value)**
Dull	40.00% (n = 30, av = 2.87)	Dull	**61**.**4% (n** = **57, av** = **2**.**11)**
Oppressive	26.60% (n = 30, av = 3.17)	Oppressive	**58**.**6% (n** = **58, av** = **2**.**36)**
Palpitant	20.00% (n = 30, av = 3.37)	Palpitant	28.8% (n = 59, av = 2.95)
Pulsating	10.00% (n = 30, av = 3.63)	Pulsating	18.7% (n = 59, av = 3.31)
Sharp	**66**.**60% (n** = **30, av** = **2**.**17)**	Sharp	**62**.**7% (n** = **59, av** = **2**.**12)**
Dragging	**65**.**50% (n** = **29, av** = **2**.**21)**	Dragging	**79**.**7% (n** = **59, av** = **1**.**83)**
Hot	30.00% (n = 30, av = 3.07)	Hot	25.9% (n = 58, av = 3.05)
Burning	**63**.**30% (n** = **30, av** = **2**.**27)**	Burning	44.8% (n = 58, av = 2.64)
4b. Mental pain qualities			
**Pain quality**	**% answered “medium to high intensity” (n, average value)**	**Pain quality**	**% answered “medium to high intensity” (n, average value)**
Miserable	32.10% (n = 28, av = 3.07)	Miserable	49.2% (n = 57, av = 2.53)
Dreadful	26.60% (n = 30, av = 3.17)	Dreadful	29.8% (n = 57, av = 3.07)
Excruciating	**40**.**00% (n** = **30, av** = **2**.**87)**	Excruciating	**55**.**9% (n** = **59, av** = **2**.**46)**
Terrible	**46**.**70% (n** = **30, av** = **2**.**6)**	Terrible	**55**.**9% (n** = **59, av** = **2**.**23)**
5. Area of pain—descriptions			
**Most common answer**	**% answered (n, average value)**	**Most common answer**	**% answered (n, average value)**
5a. burning sensation			
Medium to high intensity	70.10% (n = 30, av = 3.8)	Never to low intensity	59.3% (n = 59, av = 2.95)
5b. Formication			
Medium to high intensity	83.40% (n = 30, av = 4.67)	Medium to high intensity	62.8% (n = 59, av = 3.63)
5c. Light touch is painful			
Never to low intensity	60.00% (n = 30, av = 3.1)	Never to low intensity	60.4% (n = 58, av = 2.84)
5d. Fulgurant, electrifying attacks			
Medium to high intensity	73.30% (n = 30, av = 4)	Medium to high intensity	67.3% (n = 58, av = 3.90)
5e. Sensitivity to heat or cold			
Never to low intensity	60.00% (n = 30, av = 3)	Never to low intensity	55.9% (n = 59, av = 3.41)
5f. Numbness			
Medium to high intensity	73.40% (n = 30, av = 4.37)	Never to low intensity	50.9% (n = 59, av = 3.31)
5g. Light push (with finger) painful			
Never to low intensity	60.00% (n = 30, av = 3.13)	Medium to high intensity	83.0% (n = 59, av = 4.54)
6. Pain profile, marked areas			
**Areas marked**	**% of PDs**	**Areas marked**	**% of PDs**
Dorsal and plantar side of the feet	**60**–**70%**	Vertebral column with the neck and tailbone, knee joints	**60**–**80%**
Palmar side of the fingertips	**40**–**50%**	Shoulder region, elbows, thumb saddle joint	**40**–**50%**
Dorsal side of the left palm	30%	Umbilical region, groin region, dorsal side of the knee joints, ankle joint, hand and finger joints, metatarsophalangeal joint, heel	30–40%
Lower legs	0%	parts of the ventral side of the thorax lower side of the head	0%

The study included 60 patients diagnosed with EDS. Within the EDS group, most of the patients (86.7%) indicated to suffer from pain for more than 5 years. None of the patients experienced pain for less than a year. Almost half of the patients (45%) reported pain attacks with pain in between. One third (28.3%) reported persistent pain with severe fluctuations. More than one-tenth each indicated to experience persistent pain with light fluctuations or pain attacks with pain-free episodes. In patients with pain attacks, the average attack lasted hours and occurred weekly to daily.

The study comprised 32 patients diagnosed with GBS. Within the GBS group, 75% of the patients reported to suffer from pain for more than 1 year. One quarter of the patients who completed the questionnaire reported suffering from pain for more than 5 years. The majority (34.4%) experienced pain attacks with pain-free episodes. While one quarter of the patients reported persistent pain with light fluctuations, 6.3% reported persistent pain with severe fluctuations. In 21.9% of the patients, pain attacks occurred with pain in between. In patients with pain attacks, the average attack lasted hours and occurred several times a day.

### Pain drawings

**Fig. 1:**
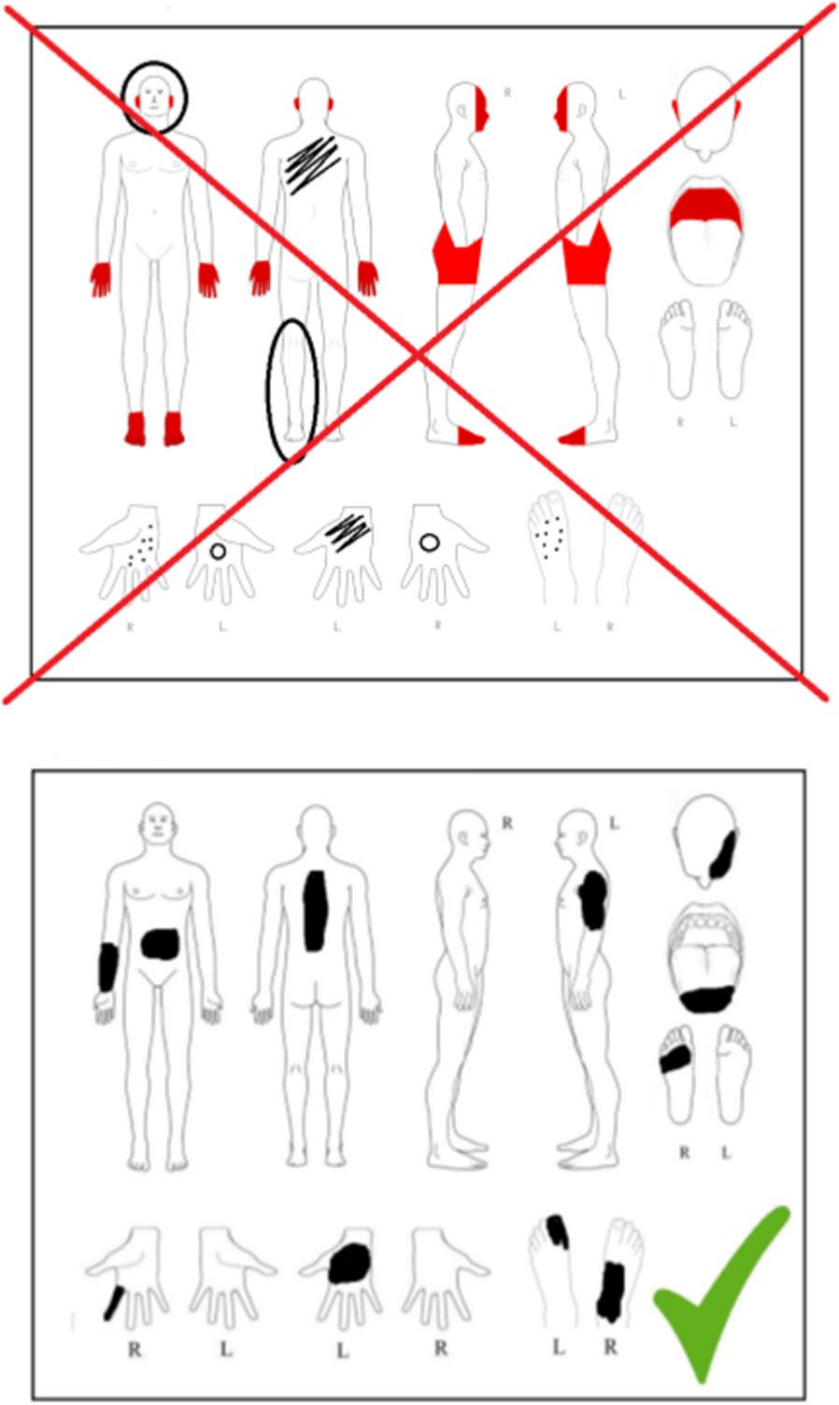
Instruction sheet handed out to patients for information on how to fill in the pain drawing

**Fig. 2:**
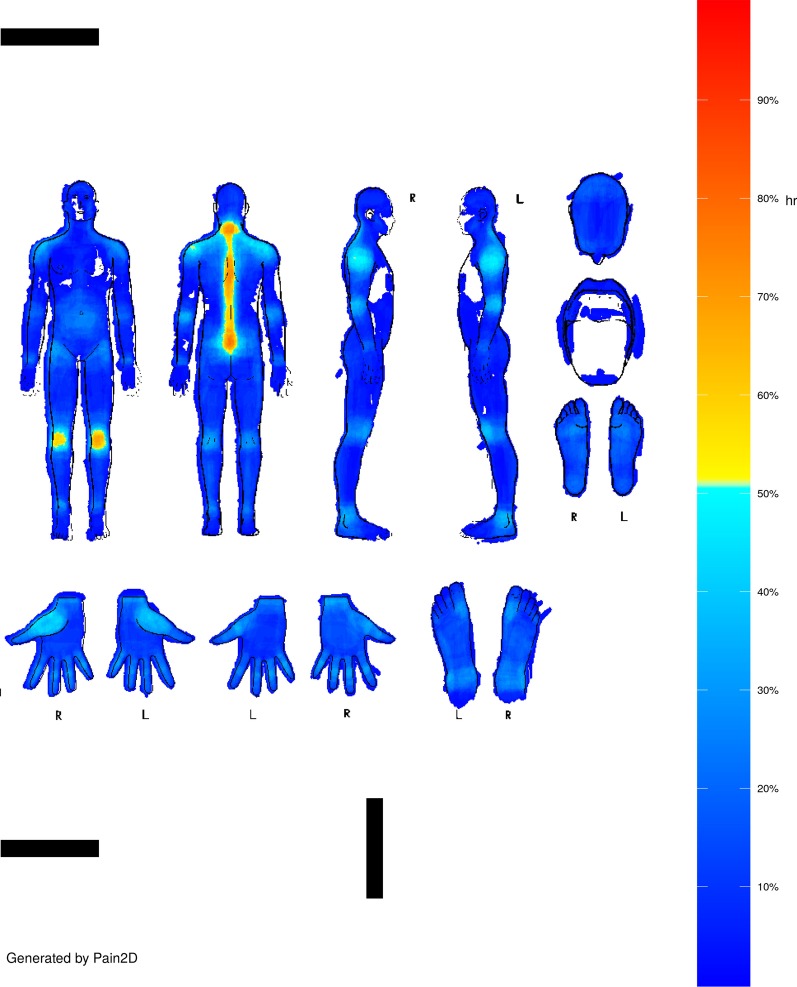
Pain profile of EDS generated by Pain2D [18]

**Fig. 3:**
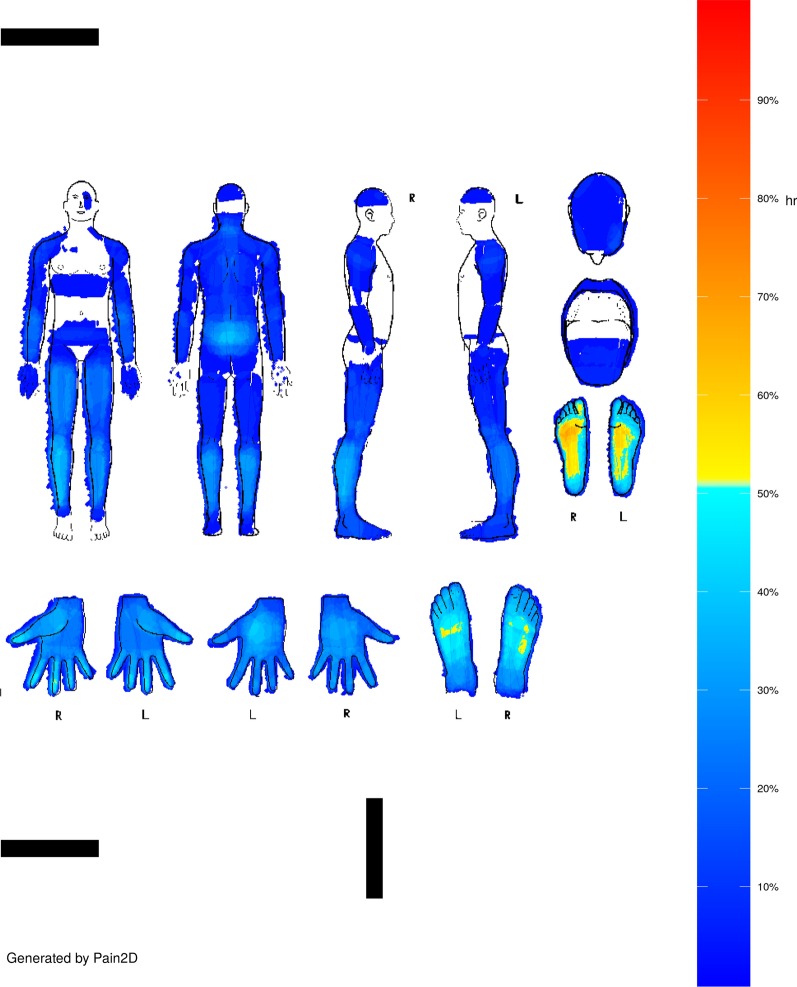
Pain profile of GBS generated by Pain2D [18]

PDs were filled in by marking the painful areas in black in the body outline sheet shown in Fig. 1, and pain profiles (PP) were generated with our newly developed software Pain2D by overlapping all PDs of one disease (s. Methods section, [18]). The PP generated by the software Pain2D is depicted as a color code image, with blue colors indicating areas less often marked as painful and red areas more often marked as painful in PDs. Pixels that were marked not at all are depicted in white. The pain profiles of EDS and GBS are depicted in Figs. 2 and 3.

#### *PainprofileEDS*

As depicted in Fig. 2, the most distinctive body regions which are painful for patients with EDS are the vertebral column with the neck and the tailbone, and the knee joints. These regions were marked by approximately 60–80% of the participating patients. About 40–50% of the participants marked the shoulder region, the elbows, and the thumb saddle joint. Only about 30–40% marked the umbilical region, the groin region, the dorsal side of the knee joints, the ankle joint, hand and finger joints, the metatarsophalangeal joint, and the heel. Focusing on the joint regions, the disease pattern we obtained reflects the characteristic pain known in patients with EDS (Fig. 2).

#### *PainprofileGBS*

Within the GBS group some regions of the body, like parts of the ventral side of the thorax and the lower side of the head, were marked by none of the participants (Fig. 3, white areas). Distinctive body regions marked by approximately 60–70% of the patients are the dorsal and plantar side of the feet. The palmar side of the fingertips, the dorsal side of the left palm, the lower legs, and the tailbone was marked by around 40–50% of the participants. The thumb saddle joint, and the dorsal side of the right palm were marked by up to 30% of the participants.

### EDS and GBS disease prediction

For the specific differentiation between both rare diseases, there has been no reference standard. We used the binary classifier of the newly developed software Pain2D [18] which works with a leave-one-out cross validation approach. It compares each PD to the pain profiles and assigns a diagnosis based on Ružička similarity (s. Methods section). The similarity between PDs is characterized by the marked pain points and not by the empty regions. Therefore, the Jaccard index would be the obvious choice to measure similarity between pain drawings. However, since the similarity between a PD and a PP had to be measured and PPs are non-binary, Ružička similarity is the obvious choice as the extension of the Jaccard index from binary to continuous objects [19].

**Fig. 4:**
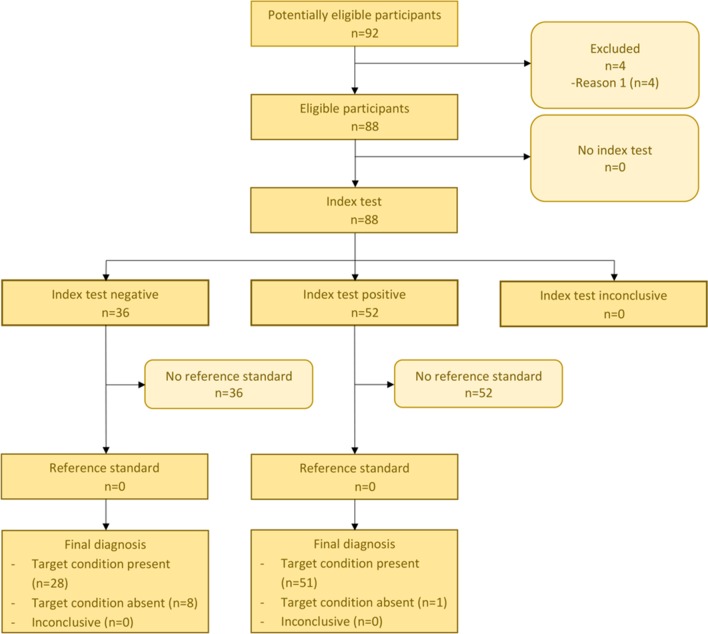
STARD diagram for EDS

**Fig. 5:**
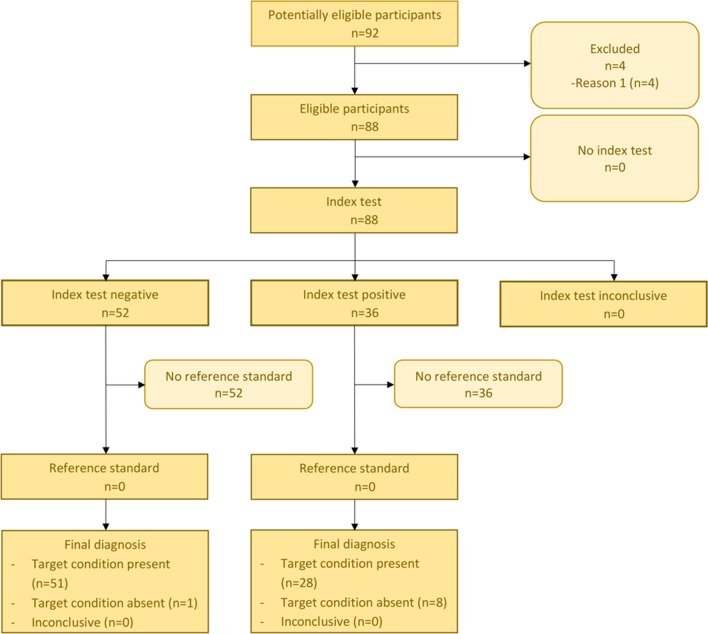
STARD diagram for GBS

The confusion matrix (Table 2) shows that 51 from 59 (86%) of the participants who suffered from EDS were correctly identified from PDs by computing the Ružička similarity coefficient to the EDS and the GBS pain profile, while only 8 participants were classified to a wrong disease (GBS). 28 (96%) PDs from GBS were classified correctly, while only one PD was wrongly classified (Table 2, Figs. 4, 5).

**Table 2:** Confusion matrix for EDS and GBS generated by the binary classifier of Pain2D

		Known disease	
		EDS	GBS
Test outcome	EDS	51	1
	GBS	8	28

**Fig. 6:**
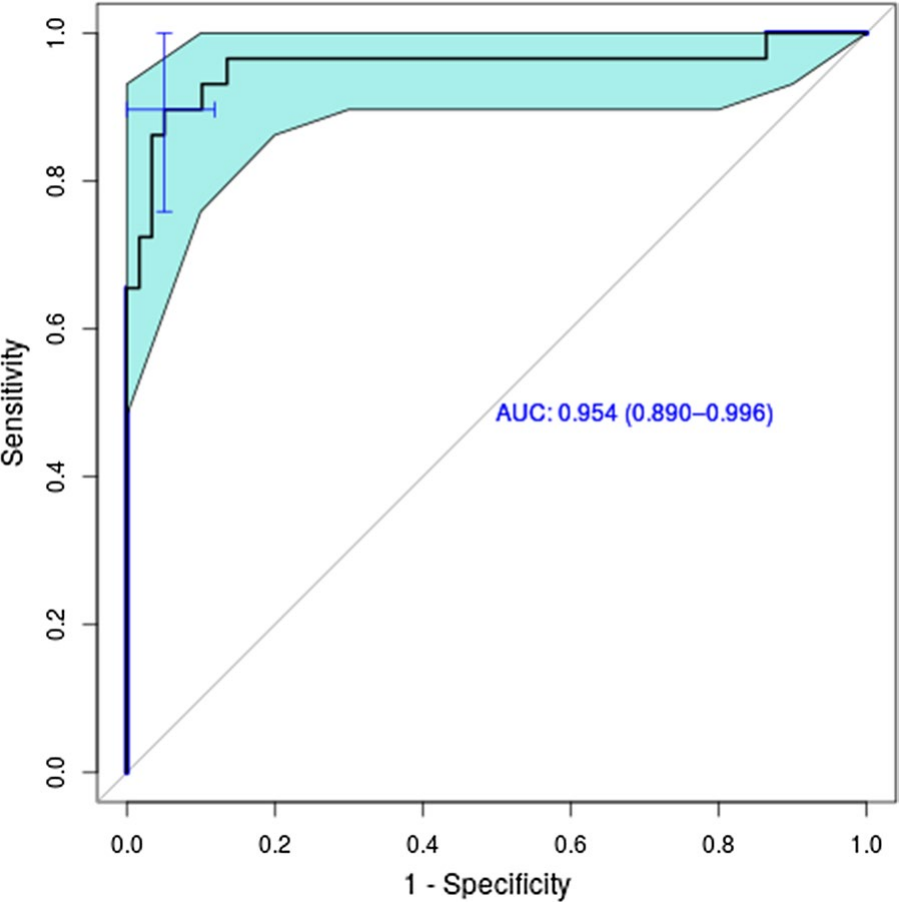
ROC curve for the classification result. The light blue area indicates confidence intervals. AUC value (confidence interval) is depicted in blue. The optimal cut-off value of 0.532 with sensitivity 94.9% and specificity 89.7% is marked by the blue crosshair

The standard cut-off value of the binary classifier, i.e., the value at which the decision between EDS and GBS classification was cut off, was set to 0.5, which gave the results summarized in Tables 2 and 3, and Figs. 4 and 5. To analyze the performance of our binary classifier with different cut-off points and to determine how well it discriminates between two classes, a receiver operating characteristic (ROC) curve was computed (Fig. 6) with the pROC package from R [20]. The ROC curve plots the true positive rate (sensitivity) against the false positive rate (1-specificity) for all possible cut-off values [21]. The ROC curve analysis showed excellent accuracy to distinguish between patients who suffer from EDS and patients who suffer from GBS. It showed a compellingly high area under the curve (AUC) value of 95% (Fig. 6). The 95% confidence band (light blue area, Fig. 6) was computed for sensitivity. The result showed a low variance for each cut-off point. The best cut-off point was proposed by pROC and had a value of 0.532 (Fig. 6, marked by crosshair) with sensitivity 94.9% and specificity 89.7%, and a misclassification rate of 7%. This proposed optimal cut-off point is close to our standard cut-off point of 0.5, with which we achieve a misclassification rate of 10% with sensitivity 86% and specificity 96% (summarized in Tables 2, 3).

**Table 3:** *p* value (Fisher’s exact test), accuracy, sensitivity, specificity, positive and negative predictive value (PPV, NPV) of the confusion matrix (Table 2), and area under the curve (AUC) for the receiver operating characteristic (ROC) curve depicted in Fig. 6

	*p* value	Accuracy	Sensitivity	Specificity	PPV	NPV	AUC
EDS.GBS	1.046e−14	0.9	0.86	0.96	0.98	0.78	0.954

The 95% bootstrap confidence interval for AUC [20] was 0.887–0.996. Fisher’s exact test was applied to the confusion matrix (Table 2) to test whether the classifier performed better than random assignment of the diseases. The *p* value suggested strongly that the classifier performed much better than random guessing (Table 3).

## Discussion

Rare diseases as a group are one of the major, but often overlooked challenges for modern health care systems. In Germany alone, around 4 million patients suffer from rare diseases, and it is estimated that approximately 350 million people are affected worldwide. Approximately 7000 rare diseases have been identified so far [22, 23]. Rare diseases are difficult to diagnose due to their heterogeneity and their complex symptoms. As a result of their rarity and diversity, most clinicians have neither the time nor the specific knowledge to provide patients with a fast and reliable diagnosis [4], and even if a rare disease is suspected, misdiagnosis is possible due to the often overlapping symptoms of many rare diseases [4]. Therefore, the average time from the first symptoms to the correct diagnosis is 6 years, but many patients have to wait far longer, up to decades [24, 25]. During this time, patients often receive several wrong diagnoses, undergo wrong treatments and suffer not only from their symptoms but also from “frustration” and “self-doubt” [26]. Diagnostic delays consequently add to the burden of individual affected patients as well as health care systems in general. Therefore, as stated by the European Union Committee of Experts on rare diseases (EUCERD), diagnosis must be one priority area in the field of research on rare disease, and there is a clear need for improving the diagnostic process [27].

Ehlers–Danlos syndrome (EDS) and Guillain-Barré syndrome (GBS) are two rare diseases associated with pain, but with dissimilar clinical presentation and etiology. EDS is a heritable connective tissue disorder with a number of subtypes, and has an estimated prevalence of 1 in 5000. EDS is characterized by joint hypermobility, skin hyperextensibility and tissue fragility with bruising, and definitive diagnosis is verified by genetic testing, with exception of the hypermobile EDS subtype, for which the genetic basis is unknown [9]. GBS is an immune system mediated disease affecting peripheral nerves and the nerve roots. It is most often triggered by infections, and typically presents initially with bilateral weakness of the legs and arms. Diagnosis is based on clinical features, electrophysiological studies and cerebrospinal fluid analysis [15, 16]. The rationale to choose these two diseases was the presence of distinct diagnostic criteria for both diseases, which allowed us to evaluate our tool by comparing its results to a previously verified diagnosis and to demonstrate the feasibility of a rare disease classifier based on the so far underutilized tool of pain drawings.

By collecting PDs from patients with the previously verified diagnosis EDS or GBS, and averaging all PDs from each disease, we obtained a specific disease pattern for EDS and GBS, the pain profile (PP). Using the Ružička similarity coefficient, the binary classifier from Pain2D [18] was able to classify each PD as EDS or GBS with high accuracy. Within the EDS group, 86% of the PDs (51 out of 59) were identified correctly. PDs from patients with GBS were classified correctly in 96% of the cases (28 out of 29). The very high AUC value of 0.95 of the ROC curve analysis confirms the high accuracy for the distinction between EDS and GBS.

As chronic pain occurs in one of five patients with rare diseases, and is frequently the first symptom that leads to medical consultation [28], pain assessment has a considerable potential for the diagnosis of rare diseases. Shaballout et al. recently showed that electronic PDs can indeed enhance physicians’ insight into acute pain sensation and improve pain communication [29], and the excellent results of the binary classifier of Pain2D for EDS.GBS classification highlights the diagnostic potential of the regional information contained in PDs and demonstrates the accessibility of this data type for computer based diagnostic aid tools.

To our knowledge, Pain2D [18] is the first attempt to develop a diagnostic aid tool utilizing information about pain, although a number of questionnaires, like “painDETECT” [30], “German pain questionnaire” [31], “Numeric Pain Rating Scale” [32, 33], “Visual Analog Scale” [34, 35], and “McGill Pain Questionnaire” [36], are available in order to describe, classify and detect different sorts of pain like neuropathic and nociceptive pain and distinguish between acute and chronic pain. A diagnostic interpretation algorithm or their evaluation as a diagnostic tool for (rare) diseases is lacking. However, there are a few studies evaluating the diagnostic potential of PDs, but without the development of specific computer aided diagnostic tools. Rennerfelt et al. used PDs for the diagnosis of chronic anterior compartment syndrome (CACS) in patients with exercise-induced leg pain [6]. Classification of individual drawings was done by two human observers. They reported a sensitivity of 67% (observer 1) and 75% (observer 2) and specificity of 65% and 54%. Another study investigated PDs as a tool for the diagnosis of single level lumbar disc herniation and reported an accuracy of ~ 68.8% [37]. Both studies achieved lower values than our EDS.GBS classifier, with an accuracy of 90%, sensitivity of 86%, and specificity of 96%.

Comparison of our results with other computer aided diagnostic tools is difficult due to the differences in approaches, as is pointed out by the authors of a recent scoping review summarizing current developments in the field of rare disease diagnosis support [38]. They identified 72 studies published between 2009 and 2019, with roughly half of those published after 2016, highlighting the very recent progress made towards this end. In the group of data driven approaches, most studies were based on image data (14 of 29 data driven studies reviewed), which is the category to which our study contributes as well. The authors comment that a comparison between studies with regard to validation and metrics is hampered by the diversity of measures used by different groups (like specificity, sensitivity, ROC and AUC, or proportion of correct diagnosis within the top k recommendations), and the differences in tool designs, diseases classified, study participants, and parameters measured. Furthermore, none of the studies included in the review dealt with pain as the main diagnostic parameter. Our tool might therefore close a gap in the current efforts towards the development of diagnostic support systems for rare diseases.

Of note, the main goal of the present study was not a new diagnostic approach to EDS and GBS, since their differential diagnoses would not normally overlap due to the clear differences in clinical presentation and etiology (although the classifier is able to distinguish effectively between the two diseases). Instead, it represents the first step towards a general rare disease classifier based on pain drawings. The excellent performance of our binary classifier for EDS.GBS classification suggests that PDs are indeed a valid tool to distinguish between rare diseases and shows that Pain2D [18] has the potential to augment diagnosis of diseases based on pain drawings, with the ultimate goal to cover the majority of pain-associated rare diseases. The procedure used in our study to create a typical pain pattern for each disease by scanning PDs and overlapping the marked areas can easily be applied to a wide range of further pain-associated diseases. Further clinical studies are currently implemented by our research group to investigate whether PDs are useful in the diagnosis of rare neurologic diseases, and we are working on the development of a k-disease classifier that includes more rare diseases. Ultimately, Pain2D [18] is supposed to grow to include many, if not most rare diseases that present with pain, at which point it potentially could indeed contribute to EDS or GBS diagnosis in a realistic clinical setting as a cheap and reliable method, and complement the clinical, genetic or lab based diagnostic parameters used right now by clinicians [9, 15, 16].

From a practical perspective in a clinical setting, Pain2D [18] in its final form is supposed to narrow down the possibilities of diagnosis to accelerate the diagnostic process. Like most computer aided diagnosis support tools, it is not meant to be a one-and-done approach, but to make diagnosis of rare diseases easier by concentrating on a few possible candidates [38]. The final diagnosis will always have to be done by other clinical methods, but the advantage of the diagnostic aid tool is that it is quick, easy, non-interventional, and can be applied in any setting where PDs can be generated. Being given a short explanation, a wide spectrum of patients was able to create PDs in a very short time without any further guidance. Only a small number of patients had difficulties because of pain in hands or fingers. Therefore, this diagnostic tool is suitable, for example, to be completed during the waiting time in outpatient clinics.

While we plan to include most rare diseases presenting with pain into future iterations of the tool, it will not be able to diagnose everything, since some diseases might have pain profiles that are more randomly distributed over the body, and it is of course unable to diagnose diseases that don’t present with pain. In addition, a limitation inherent to rare diseases is the relatively small sample size due to the small number of patients suffering from rare diseases. Furthermore, like all self-report instruments, the binary classifier has to deal with subjective interpretation of pain perception. The procedure for PD evaluation also has its limitations, since painful regions were marked using only one color. Thus, it is not possible to distinguish different qualities or intensities of pain, nor between e.g. pain of the skin at the joints, vs. joint pain. This is a limitation of the method, that for now, for the two examples used, is however not interfering with correct classification, as shown by the excellent results of the binary classifier (Table 3). A combined approach that includes evaluation of PDs and pain questionnaires in a single tool might in the future be able to increase the sensitivity by providing qualitative information about the pain experienced by patients.

Recently, Grigull et al. developed a questionnaire-based diagnostic support tool for several rare neuromuscular diseases by combining patient-oriented questions and data mining algorithms [39], a study that is also included in the recent review by Faviez et al. [38]. It is conceivable that a combination of PD analysis with this advancement in taking medical history will take diagnostic procedures of rare diseases to a new level of improvement.

## Conclusion

PDs are a useful and valid tool to distinguish between Ehlers-Danlos-Syndrome and Guillain-Barré-Syndrome. Future studies should analyze their potential in the detection of pain-associated rare diseases, as they could contribute to the optimization of diagnostic procedures in rare diseases.

## Methods

### Study design and population

The trial was designed as a prospective, observational, non-interventional study in patients with Ehlers-Danlos-Syndrome and Guillain-Barré-Syndrome. The study design and protocol were reviewed and approved by the local ethics committee, registered in the German Clinical Trials Register No. DRKS00014777 (Deutsches Register klinischer Studien, DRKS), and performed in accordance with the Declaration of Helsinki and in compliance with Good Clinical Practice. Written informed consent was obtained from each patient.

Between 2017 and 2018, a total of 60 patients with EDS (4 male, 56 female; aged 39.2 ± 11.4 years) and 32 patients with GBS (11 male, 21 female; aged 50.5 ± 13.7 years) participated in this study. To recruit patients, we contacted support groups for the diseases and attended their meetings. In addition, we used different social media platforms organized by support groups to draw attention to the study.

After informed consent patients received a questionnaire and a PD by mail or via personal contact. To be sure that the patients filled out the questionnaire and the PD properly, we added a brief explanation (see Fig. 1). PDs were scanned and automatically adjusted for shift and rotation based on the position of black bars that were printed on the PDs for this calibration purpose. Using standard image processing techniques, noise and the printed outlines of the body and body parts were removed so that only the areas marked by the patients were left [18].

#### *Inclusioncriteria*

Only patients without any other chronic disease besides the ones investigated in this study were included. Inclusion criteria were as follows: patients with GBS or EDS with a minimum age of 18 years, and written consent obtained after a detailed explanation of the investigation.

### Questionnaire and pain drawing (PD)

To evaluate different aspects of pain symptomatology, patients filled in a questionnaire based on the “painDETECT questionnaire” and the “German pain questionnaire”. Besides personal information (age, height, weight and sex), the questionnaire asked for the chronicity of pain (rating scale from less than 1 month to more than 5 years), pain course pattern (persistent pain with slight/severe fluctuations, pain attacks with pain-free episodes/pain in between), frequency of pain attacks (from several times a day to less than once a month), duration of the pain attacks (from a few seconds to more than 3 days), and dependency on time of the day. In addition, a list of eight adjectives describing the sensory, and four adjectives describing the emotional aspect of pain perception was used. Furthermore, seven questions concerning specific characteristics of neuropathic pain were included.

In addition to the questionnaire the patients received a sketch of a human body (PD) with anterior, posterior and lateral views. Additionally, head, mouth, as well as anterior and posterior views of hands and feet were magnified to receive more detailed information of these regions (see Fig. 1). Patients received also an explanation how to fill out the pain drawing.

The patients marked the afflicted areas of pain on the drawings. If the PDs for different reasons (too fine, creasing of questionnaire) lacked electronic readability, the template PD was transferred into a new PD with thicker lines or an uncreased questionnaire. This problem occurred in 10% of the PDs in the EDS group and 16% of the PDs in the GBS group.

We used the software Pain2D, which was previously developed by our group [18], to generate pain profiles (PPs) by overlapping and averaging all PDs which belong to one disease. The PP is depicted as a color code image, with blue colors indicating that a low number (below 50%) of the PDs had the corresponding pixel marked, and yellow to red colors indicating that more PDs had the corresponding pixel marked (above 50%). Green marks the 50% border. Pixels that were not marked at all are depicted in white.

### Data evaluation and statistics

In a leave-one-out cross-validation approach, each PD was classified by the binary classifier of Pain2D [18] as either EDS or GBS by calculating the Ružička similarity to both profiles. This means that one PD was removed from the data set. The remaining images were used to compute pain profiles for EDS and GBS by averaging all PDs from the corresponding disease. For the PD that was previously removed from the data set, the Ružička similarity coefficients to the EDS and the GBS average image were then computed. An EDS score—and similarly a GBS score—was also computed by dividing the EDS similarity by the sum of the EDS and the GBS similarity. Classification occurred according to the highest probability, with the cut-off set to 0.5. The whole procedure of removing a single PD was repeated for each PD. Using the computed probabilities, we plotted a ROC curve and obtained an AUC value of 0.95. In total, 28 out of 29 GBS PDs and 49 out of 59 EDS PDs were correctly classified. Fisher’s exact test and confidence intervals were calculated (as explained in more detail in the results section and figures; Table 2, Fig. 6).

### Software

NS developed a software Pain2D [18] with the open-source programming language R and RShiny. R was also used for all statistic procedures and data visualization. Pain2D allows to extract pain points from paper PDs, generating and analyzing pain profiles and generating ROC-curves and statistics with the binary classifier.

## Data Availability

All data discussed are included with the published article.

## Abbreviations

PD: Pain drawing
PP: Pain profile
EDS: Ehlers-Danlos Syndrome
GBS: Guillain-Barré Syndrome
CACS: Chronic anterior compartment syndrome
RCLBP: Recurrent or chronic low back pain
DRKS: Deutsches Register klinischer Studien
ROC: Receiver operating characteristic
AUC: Area under the curve
STARD: Standards for the reporting of diagnostic accuracy studies
PPV, NPV: Positive and negative predictive value
